# Novel rapid-acting antidepressants: molecular and cellular signaling mechanisms

**DOI:** 10.1042/NS20170010

**Published:** 2017-09-05

**Authors:** Alexandra M. Thomas, Ronald S. Duman

**Affiliations:** Department of Psychiatry, Yale University, New Haven, CT 06508, U.S.A.

**Keywords:** AMPAR, GABAR, Major Depressive Disorder, mGluR, neural circuits

## Abstract

Depression is a chronic, debilitating, and common illness. Currently available pharmacotherapies can be helpful but have several major drawbacks, including substantial rates of low or no response and a long therapeutic time lag. In pursuit of better treatment options, recent research has focussed on rapid-acting antidepressants, including the* N*-methyl-d-aspartate (NMDA) receptor (NMDAR) antagonist ketamine, which affects a range of signaling pathways in ways that are distinct from the mechanisms of typical antidepressants. Because ketamine and similar drugs hold the promise of dramatically improving treatment options for depressed patients, there has been considerable interest in developing new ways to understand how these compounds affect the brain. Here, we review the current understanding of how rapid-acting antidepressants function, including their effects on neuronal signaling pathways and neural circuits, and the research techniques being used to address these questions.

## Introduction

Major depressive disorder (MDD) affects an estimated 5% of the global population at any given time, and is the leading cause of disability worldwide [1]. In addition to the high toll of personal suffering it exacts, depression drains over $50 billion per year from the United States economy alone in lost work productivity and medical costs [2]. Despite the widespread need for effective treatment, currently available antidepressants often take 6–8 weeks to show effectiveness, and only one-third of patients respond to their first trial on any given drug. One-third of depressed patients never get relief from typical antidepressants, even after multiple trials [3]. Perhaps the biggest obstacle to the development of better medications has been the lack of understanding of the molecular mechanisms that underlie antidepressant effects. But several innovations in the past two decades have begun to reveal answers to this puzzle.

First, the drug ketamine, which had long been used in high doses as an anesthetic, was found to have a rapid antidepressant effect in low, subanesthetic doses [4]. It relieves symptoms within hours, even in many patients who have not responded to typical antidepressants. Notably, it primarily acts through a different neurotransmitter, glutamate, than do all currently available antidepressants, which primarily affect the transmission of serotonin and/or norepinephrine. The discovery of the rapid antidepressant action of ketamine and a handful of other drugs has spurred a rethinking of fundamental questions about how antidepressants work, and especially about the role of glutamate in antidepressant mechanisms. To aid in this reassessment, new tools in neuroscience have shed light on the intracellular signals and neuronal networks that underlie the effects of rapid-acting agents.

This article will review the current state of research on the mechanism of action of antidepressants, including key signaling pathways, the emerging understanding of the role of neural circuits, and the cutting-edge approaches and agents that are helping researchers understand these mechanisms.

## Brain pathology in depression

In order to understand how antidepressants relieve the symptoms of depression, it is helpful to discuss how the brains of depressed people differ from those who are not depressed. This question has been difficult to study due to the wide diversity of clinical presentations that meet criteria for MDD according to the Diagnostic and Statistical Manual of Mental Disorders (DSM) [5]. Derangements in a variety of biological processes have been imputed to lead to depression, including inflammation [6], metabolism [7], and stress-response pathways [8], and it is possible that these mechanisms interact in different ways in different subgroups of patients with MDD. Despite the probable heterogeneity of MDD mechanisms, there seem to be several common features of the depressed state that serve as hallmarks of the depressed brain.

Human neuroimaging studies have consistently demonstrated reduced brain volume in key areas associated with mood regulation, including the frontal cortex, cingulate cortex, and hippocampus [9]. Most of the volume reduction occurs in gray matter, and evidence in both humans and animals suggest that loss of glia accounts for most of this effect, and reduction in the size of neurones also plays a role [10,11]. Reduction in synapse number in the prefrontal cortex has also been found in postmortem tissue of depressed subjects and may contribute to decreased cortical gray matter volume [12]. Glial and neuronal atrophy may be a consequence of several aspects of the stress response, including excessive release of glutamate caused by high levels of corticosteroids, decreased expression of neurotrophic factors, and increased activation of apoptotic signaling pathways [13].

Glia are key regulators of glutamate neurotransmission, and their disruption leads to derangements in glutamatergic signaling that may be ameliorated by rapid-acting antidepressants. Specifically, glia inactivate glutamate signaling by sequestering glutamate after it is released into the synapse. With that function compromised, extracellular glutamate levels are elevated [14]. This excess glutamate, if present at high enough levels, will bind not only to the postsynaptic α-amino-3-hydroxy-5-methyl-4-isoxazolepropionic acid (AMPA) and *N*-methyl-d-aspartate (NMDA) receptors (NMDARs) that are its primary target, but also to presynaptic metabotropic glutamate receptors (mGluRs). Activation of these presynaptic metabotropic receptors inhibits synaptic glutamate release, which leads to reduced postsynaptic glutamatergic signaling and ultimately reduced synaptic connectivity [15]. This idea of excess glutamate leading to a reduced connectivity accords well with human neuroimaging studies, which have found elevated glutamate levels and reduced functional connectivity in the anterior cingulate cortex [16]. In addition, depressed patients have higher levels of activity in cingulate area 25, which normalizes after successful treatment with deep brain stimulation (DBS) [17].

**Figure 1:**
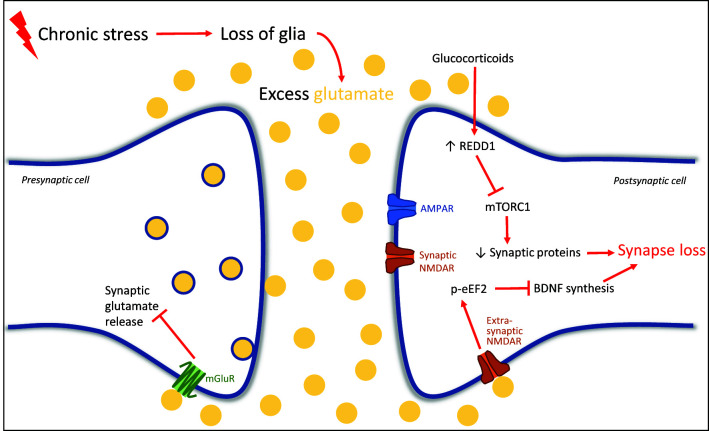
Mechanisms of synapse loss in depression Stress-induced loss of glia leads to excess extracellular glutamate, as glia normally remove glutamate from the synapse after an action potential. Glutamate then binds to presynaptic mGluRs that inhibit further synaptic glutamate release, which would normally promote strengthening of synapses by binding postsynaptic AMPA receptors (AMPARs). Glutamate binding to extrasynaptic NMDARs leads to phosphorylation of eEF2, which inhibits synthesis of BDNF, a key promoter of synaptic growth. Stress also leads to induction of REDD1, which inhibits the mTORC1 pathway. mTORC1 promotes the translation of synaptic proteins necessary for new dendrite formation. Each of these pathways contributes to the loss of synapses and dendritic spines seen in depression.

Excess extracellular glutamate may also have deleterious effects on connectivity by activating extrasynaptic NMDARs. Stimulation of these receptors initiates a signaling cascade that may be involved in the mechanism of rapid-acting antidepressants. Key components include the phosphorylation of eukaryotic elongation factor-2 (eEF2) and reduction in brain-derived neurotrophic factor (BDNF) levels, which lead to dendritic atrophy and dendritic-spine loss [14]. Induction of REDD1, a negative regulator of the mammalian target of rapamycin complex 1 (mTORC1) pathway, which is involved in synaptic protein synthesis, has been reported in postmortem PFC of depressed subjects and in rodent chronic stress models and may also contribute to the loss of synapses [18]. The degeneration of dendritic structure is a consistent finding in animal models of depression and corresponds to human studies showing loss of synapses and neuronal atrophy in MDD patients [12]. This model of glial loss leading to a decrease in connectivity and synaptic function provides important insights into the mechanism of action of rapid-acting antidepressants, which ameliorate those same deficits (Figure 1).

## Mechanism of action of currently available antidepressants

The research that would eventually lead to the development of the antidepressants in wide use today began in the 1950s, when it was noted that drugs that prevented the reuptake of monoamine neurotransmitters had antidepressant activity, though the exact mechanism remained unclear. As all these drugs increased synaptic levels of serotonin, norepinephrine, dopamine, or some combination of the three, the prevailing hypothesis was that the increase in monoamine levels was the key to their effectiveness. Based on this monoamine hypothesis, pharmacologists have been able to improve upon the monoamine-reuptake inhibitors and tricyclic antidepressants, which were the first monoaminergic antidepressants in use but which often had burdensome side effects due to their relatively non-selective binding profile. The first selective serotonin-reuptake inhibitors (SSRIs) were released in the late 1980s, and they along with selective norepinephrine-reuptake inhibitors (SNRIs) have remained the first-line agents in the treatment of depression [19].

Though the monoamine hypothesis became the basis for most drug-discovery efforts in the ensuing 40 years, it had shortcomings that were difficult to resolve before advances in the understanding of depression pathophysiology that emerged over the past two decades. Notably, the most frustrating clinical aspect of monoaminergic drugs, the 6–8-week long delay in the onset of their antidepressant activity, cannot be adequately explained by the monoamine hypothesis, given that the drugs increase monoamine availability after a single effective dose [20]. Clearly, some additional mechanism besides increased monoamine levels mediates the effectiveness of these drugs. The discovery of the rapid-acting antidepressant activity of ketamine, a glutamatergic agent, forced the field to move beyond the monoamine hypothesis to integrate what is known about deficits of plasticity and connectivity in the depressed brain and the effect of rapid-acting agents on these pathways.

## Mechanism of action of ketamine

Ketamine, the best characterized rapid-acting antidepressant, marks a dramatic improvement over monoaminergic agents not only because of its speed of onset but because it relieves symptoms of depression even in patients who have not responded to other modalities, even including those who do not respond to electroconvulsive therapy and are considered treatment resistant [21]. However, it does have drawbacks that limit widespread use. Specifically, it produces dissociative and psychomimetic side effects in the immediate post-administration period (1–2 h) in a substantial proportion of patients [22], and it has abuse potential (especially in higher doses) [23]. Even more concerning, users of frequent, high doses of ketamine suffer cortical atrophy and neurotoxicity as assessed by MRI [24]. In order to harness the impressive antidepressant profile of ketamine, it is important to understand how it functions in the brain in order to apply that knowledge to develop new therapies that are safe for widespread use.

**Figure 2:**
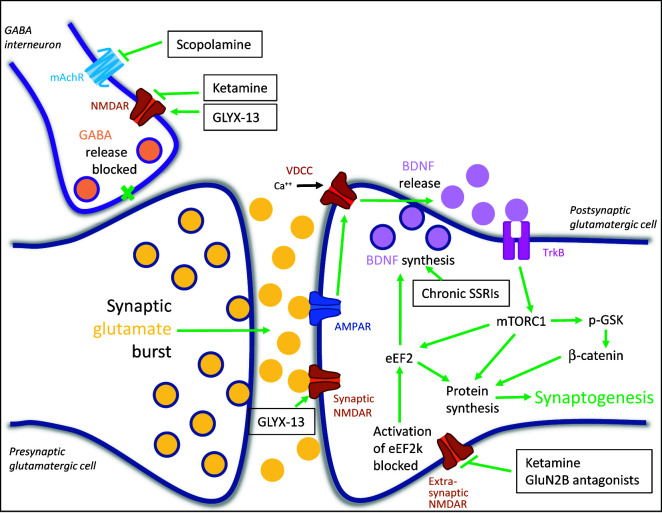
Signaling pathways involved in the response to rapid-acting antidepressants *In the GABA interneurone*: ketamine blocks the activity of the NMDAR, and scopolamine blocks the activity of the muscarinic acetylcholine receptor (mAchR); both are hypothesized to have the effect of blocking GABA release on to glutamatergic cells, which disinhibits their firing, resulting in a transient burst of glutamate release. *In the postsynaptic cell*: the glutamate burst activates synaptic AMPA receptors (AMPARs), resulting in depolarization that triggers the opening of voltage-gated calcium channels (VDCC); the resulting calcium influx triggers the release of BDNF, which binds to tropomysin receptor kinase B (TrkB) and induces mTORC1 signaling. Chronic SSRI administration increases the expression, but not activity-dependent release, of BDNF. Ketamine and GluN2B-selective NMDA antagonists exert a progrowth effect by blocking extrasynaptic NMDARs, especially those containing the GluN2B subunit. This leads to activation of elongation factor 2 kinase (EF2k), which inhibits eEF2; blockade of extrasynaptic NMDARs induce BDNF synthesis and other protein synthesis via eEF2. mTORC1 promotes protein synthesis via multiple mechanisms. Protein synthesis is necessary for activity-dependent formation of new synapses, which enables the plasticity that marks a successful antidepressant response. GLYX-13 is an NMDAR modulator that acts similar to a glycine site partial agonist. It may act at NMDAR on GABA interneurones or at synaptic NMDAR on excitatory neurons.

Ketamine is an antagonist of the NMDAR, which is an ionotropic glutamate receptor and one of the most abundant transducers of glutamate signaling in the brain. Rodent studies have demonstrated that the key to its antidepressant effect is a transient burst of glutamate that it induces in numerous regions throughout the brain shortly (30–60 min) after administration, including the medial prefrontal cortex (mPFC) [25]. Blockade of AMPA receptors blocks the drug’s antidepressant effect, providing further evidence for a role of glutamate-AMPA activity [26]. The first challenge in explaining ketamine’s mechanism of action is reconciling how a drug that blocks a glutamate receptor leads to an increase in glutamate signaling. The key to this apparent paradox may be the fact that ketamine preferentially binds to the NMDAR when its ion channel is in the open conformation (Figure 2). Interneurones have a higher tonic firing rate than pyramidal neurones and thus their NMDARs are more likely to have an open channel at any given time, so it is hypothesized that low doses of ketamine preferentially bind to NMDARs on λ-aminobutyric acid (GABA) interneurones. Blockade of the NMDAR blocks the function of these inhibitory cells, which in turn disinhibits the activity of glutamatergic pyramidal cells, whose activity is tonically inhibited by interneurones [8]. This disinhibition hypothesis explains the observed glutamatergic effects of ketamine, and it also explains why ketamine does not induce a glutamate burst or an antidepressant effect at higher doses [25]: higher concentrations of ketamine are able to bind to all NMDARs and inhibit glutamate signaling not just on interneurones but on pyramidal neurones as well, which interferes with the glutamate neurotransmission necessary to achieve an antidepressant effect.

The glutamate burst induced by disinhibition of pyramidal neurones initiates postsynaptic signaling cascades that affect both local networks in the prefrontal cortex and a wide range of other brain regions to which the pyramidal neurones project. The primary target of synaptic glutamate is the postsynaptic AMPA receptor; if AMPA receptors are inhibited, ketamine’s antidepressant effect is blocked as well [26]. AMPA receptor activation causes its ion channel to open and depolarizes the postsynaptic cell. In turn, depolarization leads to the opening of L-type voltage-gated calcium channels (VDCCs), which promotes the release of BDNF [27], binding of BDNF to its receptor tropomysin receptor kinase B (TrkB), and TrkB-mediated activation of the mTORC1 signaling pathway [28,29]. Each of these molecular signals is necessary for the antidepressant action of ketamine and ultimately promotes the dendritic-spine growth and synaptic plasticity that are the hallmarks of ketamine-induced antidepressant activity.

The signaling cascades that lead to and proceed from BDNF release and mTORC1 activation are dense and interconnected, as each is involved in different facets of the regulation of energy metabolism and cellular growth (Figure 2) [30]. Several important mediators of these pathways have been identified and their relevance to the antidepressant effect of ketamine confirmed. Autry and colleagues [31] have shown that ketamine promotes the induction of BDNF synthesis in hippocampus through an additional mechanism by preventing the activation of eEF2 kinase (eEF2K), which normally phosphorylates its target protein, eEF2, in response to spontaneous synaptic glutamate release (as distinct from action potential evoked release). NMDARs bind to spontaneously released glutamate and trigger the activation of eEF2K, so the blockade of NMDARs by ketamine prevents the transmission of this signal. Because phosphorylated eEF2 inhibits BDNF synthesis, ketamine’s NMDA antagonism removes this inhibition [32]. This effect of NMDAR antagonism is distinct from the pyramidal-cell disinhibition hypothesis, but may represent a complementary mechanism. In contrast with our previous studies as well as reports from multiple other research groups [28,33], Autry and colleagues [31] as well as a few others [34] have reported no effect of ketamine on mTORC1 signaling. This contradiction may be due to multiple factors, including uncontrolled stress of the animals, species (rat compared with mouse), brain region and dissection, and tissue preparation (crude homogenates compared with synaptosome-enriched preparations), that could influence the phosphorylation of mTORC1 signaling proteins, a process that is dynamic and state dependent.

Further supporting the idea that ketamine derives at least part of its antidepressant efficacy by blocking the response to spontaneous glutamate release, numerous studies have investigated an important role of NMDARs containing the GluN2B subunit, which is selectively activated by spontaneous glutamate release (in contrast with GluN2A subunits, which respond to action potential evoked glutamate). Pharmacological studies report that GluN2B-selective antagonists produce rapid antidepressant effects in depressed patients [35] and in rodent models [26,28]. Using a conditional knockout to remove the GluN2B subunit selectively from cortical pyramidal neurones, Hall and colleagues found that GluN2B-selective inhibition produces a robust antidepressant response that occludes the antidepressant effect of ketamine; however, these knockout mice also display hyperlocomotor activity making it difficult to interpret these behavioral findings [36]. In addition to activating in response to different patterns of glutamate release, GluN2B subunits transmit a different set of intracellular signals than GluN2A subunits and may be most prevalent at a different part of the postsynaptic neurone [37]. GluN2B-mediated signals, particularly at extrasynaptic NMDARs, appear to act as a brake on the plasticity promoting effects of glutamate neurotransmission. The conditional knockout of GluN2B removes this impediment to BDNF synthesis and mTORC1 activation in a way that occludes the effects of ketamine on both the signaling pathways [36]. Though ketamine does not selectively bind to one GluN2 isoform over the other, inhibition of overactive extrasynaptic NMDARs that contain GluN2B may have a unique set of behavioral consequences.

Ketamine also interacts with yet another facet of the plasticity regulating machinery through the glycogen synthase kinase (GSK) pathway (Figure 2). GSK controls the degradation of β-catenin, which is a necessary substrate for most forms of cellular growth and plasticity, including the formation of new dendritic spines. Phosphorylation of GSK renders it inactive, thus increasing the availability of β-catenin [30]. Ketamine rapidly promotes GSK phosphorylation, and this activity is necessary for its antidepressant effect [38]. The mechanism of this effect is not clear, but it may be a downstream consequence of BDNF release, which activates Akt, a protein that phosphorylates GSK; or it may result from mTORC1 activity, which activates S6 kinase, which also phosphorylates GSK [30].

A recent line of research has called into question the conclusion that NMDA antagonism is the functional mechanism of ketamine, based on the finding that one particular metabolite of racemic (R,S) ketamine, *(2R,6R)*-hydroxynorketamine (HNK), is sufficient to produce a robust antidepressant response, even though it was reported that this metabolite does not show binding affinity for the NMDAR [34]. This enantiomer of HNK does induce a rapid increase in glutamate signaling along with insertion of AMPA receptors in cell membranes, which racemic ketamine has recently been shown to do [39]. However, recent evidence from another laboratory indicates that HNK may in fact act at NMDARs, although at higher doses [40]. Nevertheless, even if HNK acts via NMDARs, the reduced side effects in rodent models indicate that it has the potential to be better tolerated by depressed patients than ketamine itself is.

Ketamine has numerous points of interaction with signaling pathways that lead to increased synaptic plasticity and dendritic spine growth via new translation of the proteins needed to form new synapses, including the AMPA receptor subunit GluA1 [41]. Rodent models of depression induced by chronic stress have shown that loss of dendritic spines is a key feature of the depressed brain, which ketamine reverses within 24 h of administration [42]. Both BDNF release and mTORC1 activation, two of the necessary components of ketamine’s antidepressant effect, promote synaptogenesis [43]. The restoration of synaptic plasticity appears to be the critical mechanism on which the many signaling pathways affected by ketamine converge.

## Mechanisms of action of other rapid-acting antidepressants

The ways in which other antidepressants comport with or diverge from this understanding of the mechanism of ketamine can lead to a more robust understanding of the necessary and sufficient conditions to relieve depression. Several rapid-acting antidepressants have been discovered in the past 20 years, some with efficacy similar to that of ketamine. All seem to share the key downstream mechanism of synaptogenesis and enhanced plasticity, but they achieve this effect in a variety of ways (Figure 2).

Most similar to ketamine are several agents that bind to NMDARs in different ways. As discussed above, the GluN2B selective modulators are reported to have antidepressant actions in depressed patients [35] and in rodent models [26,28]. Further clinical studies are needed to determine how efficacious these agents are in depressed patients. Another interesting compound is GLYX-13, a tetrapeptide derived from an antibody made against the NMDAR. GLYX-13 is an allosteric modulator of the NMDAR complex and has properties similar to that of a glycine-site partial agonist. It shows a rapid antidepressant effect with fewer side effects than ketamine, probably because it acts as a modulator and has a more specific binding profile. GLYX-13 also increases mTORC1 signaling and synaptic number and function in the PFC, similar to ketamine [33]. GLYX-13 may affect glutamate signaling indirectly via NMDARs on GABA interneurones, similar to ketamine, or it may act directly at postsynaptic NMDARs to enhance synaptic plasticity. Studies are currently being conducted to address this question. GLYX-13 has the potential to be better tolerated and thus more widely used as a rapid-acting antidepressant than ketamine, and clinical trials are underway to more thoroughly assess its overall effectiveness [35].

Scopolamine is a muscarinic acetylcholine receptor (mAchR) antagonist that has been found to have a rapid antidepressant effect in humans at very low doses, 4 µg/kg [44]. Like ketamine, scopolamine up-regulates mTORC1 signaling and new dendritic spine formation in the mPFC [45]. Interneurones are known to express mAchRs, and blockade of these receptors may disinhibit pyramidal cells in a similar way to ketamine’s blockade of NMDARs [39]. Using viral-mediated knockdown of the M1-AchR specifically in either GABAergic or glutamatergic cells, Wohleb and colleagues [46] demonstrated that only the M1-AChRs on GABAergic interneurones were necessary for the antidepressant effect of scopolamine.

It is also worth noting that ketamine has some molecular effects in common even with monoaminergic antidepressants, suggesting that those agents may utilize similar plasticity dependent mechanisms but in an indirect, less efficient way that cause them to exert an antidepressant effect much more slowly. In mice that have been genetically modified to express a mutated form of BDNF that impairs its expression, the antidepressant effects of both ketamine [47] and fluoxetine [48], an SSRI, are blocked. The difference is the mechanism by which these agents influence BDNF: typical antidepressant agents increase the expression but not the release of BDNF, and this occurs only after chronic administration of at least 2 weeks [49,50]; ketamine causes rapid, activity-dependent release of BDNF [27] as well as increased expression of BDNF [31] after a single dose.

## Neural circuits involved in the function of rapid-acting antidepressants

As the intracellular signaling pathways activated by antidepressants come into sharper and more detailed focus, the circuit level effects of antidepressants are beginning to be understood, thanks to new tools like optogenetics that enable the manipulation of specific brain circuits. The mood-regulating parts of the human brain have long been studied as an interrelated cortical-limbic system, and research efforts have identified correlates of these areas in non-human primates and rodents [51]. A key regulator of the limbic system is the mPFC, which exerts top-down influence over other emotion-related areas. In humans, the mPFC is thought to be involved in self-evaluation and other self-referential activities, including emotional ones [52]. Depression causes marked deficits in self-evaluation, including feelings of guilt and worthlessness, which may stem from prefrontal dysfunction. This involvement of the mPFC in depression has been studied extensively in the field of DBS research, in which permanent electrodes are placed within brain tissue and set to continuously stimulate at a high frequency in order to relieve depression and other cognitive and affective symptoms. The most consistently effective electrode placement has proven to be the subgenual cortex, an mPFC area that is overactive in depressed patients compared with controls as assessed by fMRI [53,54]. DBS inactivates targetted axons by depleting the presynaptic neurotransmitter pool; when delivered to the cortex, it reduces the excess glutamate associated with depression [55].

It has been shown recently that optogenetic stimulation of glutamatergic neurones in the mPFC of rats, with a time course and intensity similar to that of ketamine, produces a robust and long-lasting ketamine-like synaptic and antidepressant behavioral response. Further, infusion of ketamine directly into the rat infralimbic cortex, thought to be a correlate of the human mPFC, was sufficient to produce an antidepressant effect similar to what is achieved when the drug is given systemically, and neuronal silencing of infralimbic PFC blocks the effect of systemic ketamine [56]. These studies demonstrate the critical role of glutamatergic neurones in the mPFC to the antidepressant effect of ketamine.

Further studies have begun to examine the importance of glutamatergic projections from the mPFC to other brain regions, including the dorsal raphe nucleus (DRN) and hippocampus, given that pyramidal neurones in the mPFC send axons to many other regions of the brain. The mPFC to DRN projection has been investigated using optogenetic stimulation of axon terminals in the DRN along with simultaneous behavioral analysis. These studies showed that activation of this projection produces a real-time antidepressant effect in rats [57]. In preliminary studies, we have found that stimulation of PFC terminals in the DRN also produces a more long-lasting antidepressant response in the FST 24 h after optogenetic stimulation [58]. Additional studies demonstrated that depletion of serotonin blocks the antidepressant effect of mPFC-injected ketamine, and that ketamine in the mPFC induces neuronal activation in the DRN as assessed by cFos expression [59]. Together, these studies suggest that activation of the mPFC to DRN projection may be necessary for ketamine’s antidepressant effect.

The ventral hippocampus has also been found to be relevant to the antidepressant effect of ketamine. A recent study used DREADDs (designer receptors exclusively activated by designer drugs) to mimic ketamine’s antidepressant effect by stimulating the ventral hippocampus to mPFC pathway. Further, the pharmacological or optogenetic inactivation of this pathway was found to block ketamine’s effect [60]. These results are consistent with studies showing that mTORC1 and BDNF are both up-regulated in the rat hippocampus as well as PFC after ketamine administration, indicating that ketamine produces plasticity enhancements in the hippocampus that are similar to what has been reported for the PFC [61]. Though a complete picture of the neural circuitry underlying ketamine’s effect has yet to fully emerge, these studies offer the first glimpses into the brain region-specific and circuit-specific effects of ketamine-induced plasticity.

## Future directions

The scientific understanding of the mechanisms of antidepressants has progressed tremendously in nearly two decades that have passed since the discovery of rapid-acting antidepressants and the key role of glutamate. The intracellular signals that underlie the action of rapid-acting antidepressants have been elucidated, but gaps remain. In the near future, more precise and nuanced genetic modifications in rodents may allow researchers to test hypotheses about the progression from NMDA antagonism, to BDNF release and mTORC1 activation, to synaptogenesis in the action of ketamine. There is even more room for discovery related to the specific cell types that mediate these effects and how they affect their local circuitry as cell-type-specific viral vector systems and genetic modifications become more widely available. Such approaches should yield more concrete answers about the relative importance of the pyramidal-cell disinhibition hypothesis compared with blockade of spontaneous glutamate transmission via GluN2B-containing NMDARs as ketamine’s primary mechanism of action. Finally, advances in optogenetic and chemogenetic techniques are providing new insights into the role of specific circuits in the function of antidepressants.

## Abbreviations

Akt: Akt1 serine/threonie kinase 1
AMPA: α-amino-3-hydroxy-5-methyl-4-isoxazolepropionic acid
BDNF: brain-derived neurotrophic factor
cFos: Fos proto-oncogene
DBS: deep brain stimulation
DRN: dorsal raphe nucleus
eEF2: eukaryotic elongation factor-2
eEF2K: eukaryotic elongation factor-2 kinase
fMRI: functional magnetic resonance imaging
FST: forced swim test
GABA: λ-aminobutyric acid
GABAR: λ-aminobutyric acid receptor
GluN2B: glutamate ionotropic receptor NMDA subunit 2B
GSK: glycogen synthase kinase
HNK: *(2R,6R)*-hydroxynorketamine
mAchR: muscarinic acetylcholine receptor
MDD: major depressive disorder
mPFC: medial prefrontal cortex
mTORC1: mammalian target of rapamycin complex 1
NMDA: *N*-methyl-d-aspartate
NMDAR: *N*-methyl-d-aspartate receptor
PFC: prefrontal cortex
REDD1: regulated in DNA damage and repair 1
S6: p70 ribosomal S6 kinase
SSRI: selective serotonin-reuptake inhibitor
TrkB: tropomysin receptor kinase B
